# SPECT Functional Neuroimaging Distinguishes Adult Attention Deficit Hyperactivity Disorder From Healthy Controls in Big Data Imaging Cohorts

**DOI:** 10.3389/fpsyt.2021.725788

**Published:** 2021-11-24

**Authors:** Daniel G. Amen, Theodore A. Henderson, Andrew Newberg

**Affiliations:** ^1^Amen Clinics, Inc., Costa Mesa, CA, United States; ^2^The Synaptic Space, Denver, CO, United States; ^3^The International Society of Applied Neuroimaging, Denver, CO, United States; ^4^Neuro-Luminance, Inc., Denver, CO, United States; ^5^Dr. Theodore Henderson, Inc., Denver, CO, United States; ^6^Marcus Institute of Integrative Health, Thomas Jefferson University, Philadelphia, PA, United States

**Keywords:** brain SPECT, ADHD, comorbidity, single photon emission computed tomography, inattention

## Abstract

**Background:** The diagnosis of attention deficit hyperactivity disorder (ADHD) relies on history and observation, as no reliable biomarkers have been identified. In this study, we compared a large single diagnosis group of patients with ADHD (combined, inattentive, and hyperactive) to healthy controls using brain perfusion single-photon emission computed tomography (SPECT) imaging to determine specific brain regions which could serve as potential biomarkers to reliably distinguish ADHD.

**Methods:** In a retrospective analysis, subjects (*n* = 1,135) were obtained from a large multisite psychiatric database, where resting state (baseline) and on-task SPECT scans were obtained. Only baseline scans were analyzed in the present study. Subjects were separated into two groups – Group 1 (*n* = 1,006) was composed of patients who only met criteria for ADHD with no comorbid diagnoses, while a control group (*n* = 129) composed of individuals who did not meet criteria for any psychiatric diagnosis, brain injury, or substance use served as a non-matched control. SPECT regions of interests (ROIs) and visual readings were analyzed using binary logistic regression. Predicted probabilities from this analysis were inputted into a Receiver Operating Characteristic analysis to identify sensitivity, specificity, and accuracy.

**Results:** The baseline ROIs and visual readings show significant separations from healthy controls. Sensitivity of the visual reads was 100% while specificity was >97%. The sensitivity and specificity of the *post-hoc* ROI analysis were both 100%. Decreased perfusion was primarily seen in the orbitofrontal cortices, anterior cingulate gyri, areas of the prefrontal cortices, basal ganglia, and temporal lobes. In addition, ROI analysis revealed some unexpected areas with predictive value in distinguishing ADHD, such as cerebellar subregions and portions of the temporal lobes.

**Conclusions:** Brain perfusion SPECT distinguishes adult ADHD patients without comorbidities from healthy controls. Areas which were highly significantly different from control and thus may serve as biomarkers in baseline SPECT scans included: medial anterior prefrontal cortex, left anterior temporal lobe, and right insular cortex. Future studies of these potential biomarkers in ADHD patients with comorbidities are warranted.

## Introduction

Attention deficit hyperactivity disorder (ADHD) is one the most costly psychiatric disorders, conservatively estimated to be around 42.5 billion USD annually (1). ADHD is also one of the most prevalent disorders in the USA with ~5.29–10% of school-aged children estimated to suffer from the disorder (1). Despite these enormous costs and the issues of administering stimulant medication to children, there remains no empirically validated means by which ADHD can be diagnosed. Objective markers of ADHD would not only improve reliability of the diagnosis but might also allow for precision medicine treatments (2). Problems associated with the use of subjective criteria for diagnosing ADHD are extensive (3). Subjective diagnostic criteria and related diagnostic processes are highly vulnerable to variability by different clinics and by different clinicians within the same clinic (4, 5). Moreover, even when clinicians strictly adhere to the DSM method for diagnosing ADHD, there is also significant variation in the rates of ADHD secondary to the DSM versions utilized by a clinician (6). The problem does not become easier with adult ADHD patients with whom confounds of coping strategies, substance use, and comorbidities cloud the diagnostic picture (7).

Furthermore, the cardinal symptom of ADHD—inattention—is a non-specific symptom. Inattention is found not only in ADHD, mania, anxiety, and depression, but it is also found in traumatic brain injury, carbon monoxide poisoning, cadmium toxicity, lead toxicity, schizophrenia, post-traumatic stress disorder (PTSD), post-coronary bypass syndrome, multiple sclerosis, substance abuse, space-occupying lesions, CNS infections, dementia, and a litany of other conditions which alter frontal lobe functioning. Distinguishing among these alternatives by interview alone is challenging, because, for instance, there is no specific question that will reveal lead toxicity or cadmium toxicity. Similarly, an interview may or may not uncover a history of brain injury, depending on the degree of anterograde amnesia or how the patient has trivialized the impact of a concussive event. The authors have seen numerous cases of drowning, toxicity, post-pediatric surgery, hypomania, and irritable depression which were misdiagnosed clinically as ADHD. An additional challenge in diagnosing ADHD, regardless of age, is that comorbidity is the rule, rather than the exception in ADHD.

## Diagnostic Evaluation of ADHD

The base level of precision in the diagnostic evaluation of ADHD is the use of rating scales. Measures such as the Vanderbilt Rating Scale (6), and the Conners Parent Rating Scales (8) are quantifiable, but lack diagnostic precision. Scales are dependent upon the subjective opinion of parents and/or teachers. Symptom overlap of scale items across multiple DSM diagnoses is the rule rather than the exception (9, 10).

A higher level of accuracy can be derived by the use of computerized tests of attention. While there is perception that continuous performance tests are the “objective standard” for ADHD diagnosis, the research demonstrates a distinct gap between the computerized diagnosis and the clinical presentation. For example, correlation between the Conners Continuous Performance Test (CPT) results and results of parent or teacher symptom rating scales is low to moderate (11, 12). The Test of Variables of Attention (TOVA) has a sensitivity of ~85% and a false positive rate of 30% (11, 13). In contrast, the CPT has a high false negative rate (14). Combining the continuous performance test with an infrared motion sensor (McLean Motion Attention Test or Quotient ADHD System) has been FDA-cleared as a diagnostic tool for ADHD. Using this system, Teicher et al. found that boys with ADHD moved their heads 2.3 times more often than boys without ADHD (15). However, this system is less effective in the diagnosis of inattentive-type ADHD and of adult ADHD.

## Biomarkers for ADHD

The Food and Drug Administration's (FDA) Biomarkers, EndpointS and other Tools (BEST) glossary defines a biomarker as: “a defined characteristic that is measured as an indicator of normal biological processes, pathogenic processes, or responses to an exposure or intervention, including therapeutic interventions” (16). Note that the BEST definition does not limit the nature of the characteristic to a molecule. Any characteristic can serve as an indicator of pathology or response to therapeutic intervention. A significant need remains for identifying biomarkers for psychiatric conditions, including ADHD, to provide more accurate diagnosis and to foster efforts to develop more effective treatments. While there is widespread agreement that fronto-striatal-thalamic pathways are altered in ADHD (17, 18), it has been difficult to identify a reliable neuroimaging biomarker, regardless of the neuroimaging technique.

### Quantitative EEG

Quantitative electroencephalogram (qEEG) has been FDA-approved as a diagnostic tool for ADHD and purported to serve as a biomarker. However, the marker of elevated theta/beta wave ratio is not reliably diagnostic. The pivotal study on the ratio reported a 20% false negative rate (19). Moreover, Arns et al. analyzed the collective data of more than 1,750 children and concluded that the elevated theta/beta ratio was not a reliable diagnostic measure for ADHD (20). Elevated theta/beta ratio has not proven to be the endophenotype or biomarker that was initially hoped.

### Anatomical MRI

Anatomical magnetic resonance imaging (MRI) studies have found a small number of consistent findings across the age-range of ADHD (21). Multiple meta-analyses of case-control studies have shown reduced volume of the striatum in children with ADHD (22–25); however, the reduced striatal volume in ADHD appears to correct itself with age (24, 26). Notably, reduced striatal volume is also found in children with autism spectrum disorders (27). The ENIGMA-ADHD project examined the volumes of subcortical structures in a large sample of 1,713 cases of ADHD compared to 1,529 controls (28). In children, slight, but significant, decreases in volume were found in the caudate, putamen, and amygdala, as well as the hippocampus and nucleus accumbens. However, these differences were not found in the adult subjects, confirming the results of some meta-analyses (24, 26). Similarly, the ENIGMA-ADHD analysis of cortical thickness found smaller surface areas in the frontal, cingulate, and temporal cortices in children, but not in adults (29). Further analysis of the ENIGMA data including 2,271 cases of ADHD and 5,827 controls found cortical thickness was smaller in orbital frontal, inferior frontal and cingulate cortices across all age ranges, including adults.

One limitation of this anatomical MRI research is that a majority of the studies do not control for comorbidities or medication use. Also, the relative paucity of longitudinal studies precludes determining if the brain volume changes represent a persisting difference or a delay in maturation. An additional limitation of this work is the high degree of variability within groups for any given metric (27). Lastly, while the ENIGMA-ADHD database represents an impressive feat of cross-site coordination and data collection, the ADHD population captured therein is incompletely characterized. For example, comorbidities are known in only 58% of the population and stimulant use is documented for only about half of all cases (27).

### Functional MRI

ADHD has been the subject of intense study using functional MRI over the past 24 years, yet the results have been highly divergent (30–32). Multiple meta-analyses have yielded mixed results. To quote the authors of a recent meta-analysis of 96 studies with over 1,914 subjects which found no statistically significant functional abnormalities in ADHD:

“*The overall findings indicate a lack of regional convergence in children/adolescents with ADHD, which might be due to heterogenous clinical populations, various experimental design, preprocessing, (or) statistical procedures in individual publications.”* (32)

Despite the harsh criticism of the heterogeneity in the field, these authors did find a marginally significant decrease in left inferior frontal cortex activity in male children only (32). Others have found similar task-dependent inferior frontal cortex deficits, although it varies whether the right or left side is more involved (32–36). For example, Pliszka et al. found that adolescents with ADHD (*N* = 17; age 13.4 ± 1.9 yrs) failed to show increased perfusion in the anterior cingulate bilaterally and in the left orbitofrontal prefrontal cortex during an inhibitory task (Stop Signal Task) compared to 15 age-matched controls (age 13.2 ± 1.9). These authors further analyzed the ADHD subjects by comparing children who were medication-naïve and those who were not. These two subgroups did not differ in performance or functional neuroimaging findings (37). Smith et al. described similar findings in a small group of 19 medication-naïve patients (age 12.9 ± 1.9 yrs) compared to 27 healthy controls (age 14.1 ± 2.0 yrs). They found decreased perfusion in the left rostral mesial frontal cortex during one interference-type concentration task and decreased perfusion in the bilateral inferior prefrontal (right more significant than left) and temporal lobes during a switch task (34).

Efforts to explore networks either via the default mode network (DMN) or using selected kernels to identify networks of activation, have suggested that ADHD is not a disorder of isolated brain regions, but more of a connectivity disorder (38). Nevertheless, in a recent meta-analysis involving 30 studies with 1,094 subjects with ADHD and 884 Controls, no significant functional networks or areas were found to distinguish ADHD from Controls (38).

In addition, multiple areas of the cerebral cortex, including parietal and temporal regions, as well as the cerebellum, have shown decreased activity during concentration tasks in subjects with ADHD (17). Thus, in addition to the technical discrepancies in fMRI studies, the effects of age, medication use, comorbidities, and recruitment or suppression of activity in multiple areas of the brain have hampered the ability of fMRI to reveal a consistent biomarker.

### Machine Learning – Multimodal Imaging

Machine learning or artificial intelligence (AI) techniques have been applied to neuroimaging in an effort to detect patterns and findings not evident from simple statistical analysis. Numerous AI techniques, such as support vector machine, multiple kernel learning, deep belief network, convolutional neural network and others have been applied to fMRI and anatomical MRI data (39). For example, a group of 36 adults with ADHD and 36 controls underwent anatomical MRI, fMRI using a cued attention task, and diffusion tensor imaging. Twenty features were chosen from this multimodal dataset and processed in a meta-algorithm referred to as “ensemble learning techniques” (ELT). A series of training and validation algorithms followed by multiple ELT-based models yielded a number of parameters with favorable sensitivity and specificity (18). Notably, decreased activity of the right inferior frontal gyrus stood out as a strong predictor of ADHD status. The limitation of the AI work to date has been the relatively small sample sizes, the lack of consistent findings across studies (18, 39, 40) and the need for multiple time-consuming scans.

### Functional SPECT

Functional neuroimaging studies of ADHD utilizing single-photon emission computed tomography (SPECT) in children and adults have included baseline studies, cognitive challenge studies, and medication effect studies. The controlled clinical trials have been small with a range of 6–54 subjects (reviewed in Discussion). Our earliest study in this area included 54 children who met DSM-III-R criteria for ADHD compared to a clinical group of 18 children who did not meet those criteria (41). Visual, semi-quantitative reads revealed areas of increased perfusion in dorsal frontal cortices, while areas of decreased perfusion were noted in the orbitofrontal/inferior prefrontal cortices in baseline SPECT scans. SPECT scans during intellectual challenge also revealed decreased inferior prefrontal cortical perfusion (41). A smaller study by our group in 27 older adults (>50 years) who met DSM-IV criteria for ADHD, but not for major depression, revealed a similar decrease of perfusion in the orbitofrontal cortices at baseline (42). Recently, a larger, open retrospective case series of 170 patients ranging in age from adolescent to adult utilized visual reads of SPECT scans found that visual read to assess ADHD using 3D renderings yielded 83% sensitivity and 77% specificity in the diagnosis of ADHD based predominately on the finding of decreased orbitofrontal perfusion (43).

Findings across multiple studies are consistent but are they reliable and can they be used on an individual basis to predict the diagnosis of ADHD? This question is central to the ability to use a SPECT neuroimaging finding as a biomarker. Herein, we describe the first step in an analysis of a community dataset totaling over 100,000 patients. We describe the analysis of adults with ADHD free of comorbidity compared to a control group who lack any psychiatric or neurological diagnoses. Future steps will include comparisons of comorbid ADHD across age groups and predictive modeling utilizing machine learning algorithms involving iterative comparisons of data subsets to assess the predictive value of specific biomarker candidates.

## Materials and Methods

### Study Subjects

This study adhered to the STAR-D guidelines (44) (see Supplementary Material for table). This retrospective review was approved by an accredited institutional review board, IntegReview (http://www.integreview.com/). In this retrospective analysis, all study subjects were patients at Amen Clinics, Incorporated (ACI), a multidisciplinary group of psychiatric clinics that incorporates SPECT neuroimaging into diagnostic assessment and treatment (45). Methods of clinical assessment, gamma camera equipment, scan analysis software, and interpretation protocols are unified throughout the group of clinics. All subjects were drawn from the following ACI branches: Newport Beach, CA; Brisbane, CA; Fairfield, CA; Tacoma, WA; Bellevue, WA; Reston, VA; New York, NY; Atlanta, GA. Group 1 included patients seen from April 1996 to November 2013. Informed consent was obtained at the time of patient evaluation from all patients or legal guardians to allow their anonymous clinical data to be utilized for future research purposes. We identified from this clinical cohort, Group 1 (*n* = 1,006) which included persons that met the DSM-IV criteria for ADHD (46) and no other diagnoses (Table 1). The diagnosis of ADHD (inattentive, impulsive-hyperactive, combined) was determined by DSM-IV guided clinical interview, internal DSM-IV-guided symptom checklists, and a Conners Continuous Performance Test (47). The ADHD-only group was compared to a Control group who did not meet criteria for any psychiatric condition and had no history of traumatic or toxic brain injury (*n* = 129). The Control group was recruited using local advertisements in newspapers and local colleges. Each subject met the clinical criteria for a healthy brain subject based on our criteria that included the absence of current medical illnesses, brain trauma, family history of psychiatric illness, drug/alcohol abuse and no current or past evidence of behavioral or psychiatric issues as measured by a detailed clinical history, Minnesota Multiphasic Personality Inventory (MMPI) and Structured Clinical Interview for Diagnosis (SCID) for DSM-IV. The Control group recruitment and scanning study protocol was approved by Western IRB (WIRB # 20021714). All subjects were fully informed and gave their written consent.

**Table 1:** Demographic characteristics of Group 1.

**Variable**	**ADHD**	**Control**	**Statistical comparison (t, p)**
	**(*n* = 1,006)**	**(*n* = 129)**	
Age	37.7 ± 15.5	45.4 ± 16.9	2.9, 0.004
Gender (% female)	34	44	30.6, <0.001
Race (% non-Caucasian)	31	33	48.9, <0.001
Bipolar disorder	0	0	NA
Depression	0	0	NA
Dementia	0	0	NA
Brain trauma	0	0	NA
PTSD	0	0	NA
Substance disorder	0	0	NA
Schizophrenia	0	0	NA

### SPECT Neuroimaging

Brain SPECT was applied as previously described in published work using standard methods (48). To review, all patients were instructed to refrain from the use of stimulants, caffeine, ephedrine, bupropion, atomoxetine, nicotine, alcohol, illicit drugs, opiates, benzodiazepines, guarana, or steroids for 48 h prior to scanning. Other medications including psychotropic medications were not restricted. For each scan, an age- and weight-appropriate dose of technetium Tc99m-HMPAO (commercially available as Ceretec) was administered intravenously. At all clinic sites, photon emission was captured using a high-resolution Picker (Phillips) Prism 3000 triple-headed gamma camera with fan beam collimator with data collected in 128 × 128 matrices, yielding 120 images per scan with each image separated by three degrees spanning 360 degrees. A low pass filter was applied with a high cutoff. A Chang attenuation correction was performed using linear methods (49).

All images were processed using Odyssey software (Picker), with transaxial slices oriented horizontal to the AC-PC line. Coronal, sagittal, and transaxial slice images (6.6 mm apart, unsmoothed) were then rendered in the Odyssey step-20 scale, a commercial scale included in the Odyssey software package, which scales all voxels to the brain maximum and assigns each a color gradient based on its percentile of activity. Each color step represents a (not necessarily linear) five-percentile-point change in rCBF.

Baseline images were acquired in the following manner, adapted from the Society of Nuclear Medicine and Molecular Imaging Procedure Guideline for Brain Perfusion SPECT (50). Patients sat upright in a quiet, dimly lit room with open eyes, and the bolus was injected after 10 min. Patients sat for an additional 10 min post-injection. While concentration-task scans were obtained for all patients, they are not included in the current analysis and will be subject of future studies.

### Clinician Visual Rating of Regions of Interest

Methods for clinical interpretation of SPECT scans by visual read have not changed during the 17 years of patient evaluations from which these data are drawn. These methods have already been fully described in prior peer reviewed work (42). To review, 14 gross general cortical regions of interest (ROIs) in orthogonal planes were visually inspected and rated using the Mai Atlas of the Human Brain (51): the left and right prefrontal poles [medial aspect of Brodmann area (BA) 10, anterior rostral aspect of BA 12]; the left and right inferior orbitofrontal (BA 11); the left and right anterior/lateral PFC (comprised of BAs 45, 46, 47, the anterior aspect of area 9, the lateral aspect of area 10); the left and right midlateral PFC (BAs 8 and 44, and the posterior aspect of area 9); the left and right posterior frontal region (BAs 4, 6, and the anterior aspect of area 43); the left and right parietal lobes (BAs 1, 2, 3, 5, 7, 39, and 40, and the posterior aspect of area 43); and the left and right occipital lobes (BAs 17, 18, and 19). In like manner, we rated both the left and right cerebellum. In addition, seven gross subcortical ROIs were rated: the anterior cingulate gyrus (BAs 25, 32, 33, and the anterior aspect of BA 24); the left and right insula; the left and right thalami; the left and right caudate nuclei; and the left and right putamina. The following non-linear scheme was used to visually rate rCBF: activity rated above the top 95% was assigned a score of +4; 91–95% was scored +3; 86–90% was scored +2; 81–85% was scored +1; 61–80% was scored 0; 56–60% was scored −1; 51–55% was scored −2; 46–50% was scored −3; and 41–45% was scored −4. Because of the non-uniform nature of perfusion within any given ROI, each area was rated for its highest and lowest activity, and the average of the two was taken as a given ROI's final rating, resulting in a rating scale ranging from +4 to −4 in half-point intervals. Raters had minimal clinical information. Interrater reliability was not assessed for these particular groups; however, prior studies have found a kappa of 0.79 or above for all visually-read regions (42).

### *Post-hoc* ROI Analysis

All baseline scans in the two groups were subjected to *post-hoc* ROI analysis. ROI counts were derived from the anatomical regions in the AAL atlas (52), different, but closely aligned with the regions in the atlas used for visual reads. ROI included in this study were as follows: anterior cingulate, mid-orbital frontal, insula, anterior inferior temporal, middle inferior temporal, posterior inferior temporal, temporal pole, superior parietal, hippocampus, thalamus, caudate, pallidum, cerebellar regions 7b,8,9, cerebellar crus1, and cerebellar vermis. To account for outliers, T-score derived ROI count measurements were derived using trimmed means that are calculated using all scores within the 98% confidence interval (−2.58 < Z < −2.58). The ROI mean for each subject and the trimmed mean for the sample are used to calculate with the following formula:


$$\begin{array}{l} T=10^{*}((subject\;roi\_mean-trimmed\;regional\_avg)/\\ \;trimmed\;regional\_stdev)+50 \end{array}$$

### Statistical Analyses

All analyses were performed using Statistical Package for Social Science (SPSS) (53). In Group 1, a receiver operating characteristic (ROC) curve analysis was done using DSM-IV diagnosis for ADHD as ground truth. The first step of this analysis was constructing logistic regression models with age, gender, and race as co-variates. Separate models were constructed with the following independent variables: (i) Baseline visual reads, and (ii) T-score ROI counts from baseline scans. From each of these logistic regression models, odds ratios and predicted probabilities were computed and then inputted into an ROC analysis to determine area under the curve, or accuracy of the given methods used. For ROI data, one way ANOVA with Least Square Differences (LSD) for correcting for multiple comparisons was done to assess group differences. Automated linear regression was used for feature selection. Correction for multiple comparisons were performed in each logistic regression model.

## Results

The total sample of 1,135 subjects were separated into two groups. Group 1 consisted of patients who met the DSM-IV criteria for ADHD, and it contained 1,006 subjects (see Table 1). The mean age was 37.7 ± 15.5, making it somewhat younger than the control group (*n* = 129) with a mean age of 45.4 ± 16.9 (*p* ≤ 0.001). Group 1 was 34% female and 31% non-Caucasian, while the control group was 44% female and 33% non-Caucasian (*p* < 0.001 in age group and non-significant for non-Caucasian). Group 1 did not meet criteria for any other psychiatric disorder, substance abuse, or brain injury based on a detailed clinical history, the Minnesota Multiphasic Personality Inventory (MMPI) and the Structured Clinical Interview for Diagnosis (SCID) for DSM-IV.

**Figure 1:**
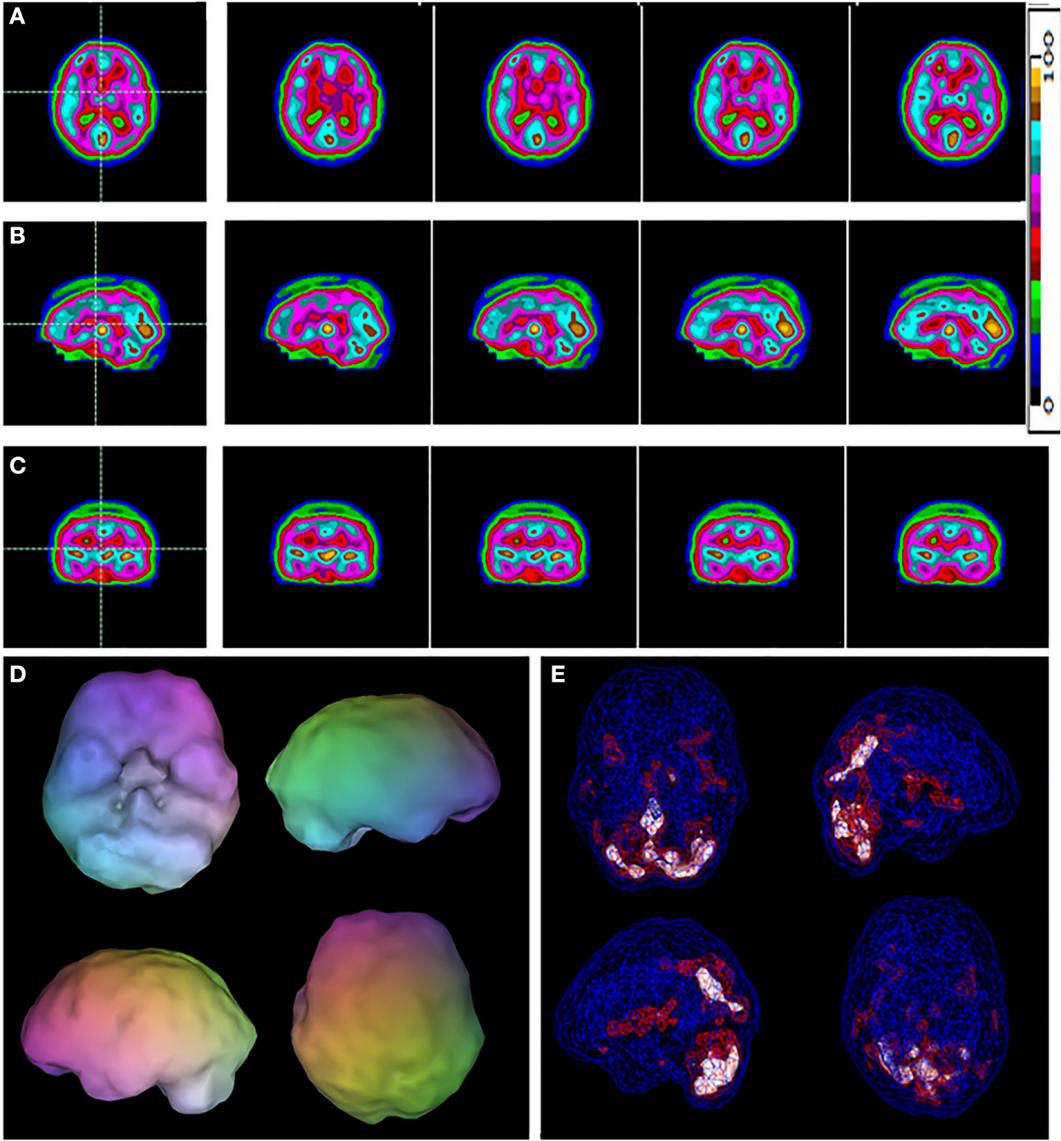
A typical example of a control case. Selected tomograms in horizontal **(A)**, sagittal **(B)**, and coronal **(C)** orientation are provided. The color scale for the tomograms is provided. All voxels are scaled to the brain maximum and assigned each a color gradient based on its percentile of activity. Each color step represents a (not necessarily linear) five-percentile-point change in rCBF. **(D)** A 3-D representation of the scan data is shown. The surface is set at 60% of brain maximum. Areas which fall below 55% are represented as indentations or holes depending on how far below 55% the activity falls. **(E)** A wireframe brain representation is shown, wherein the areas of brain with activity at 85% of maximum or greater are shown in red and areas of 92% or greater are shown in white.

**Figure 2:**
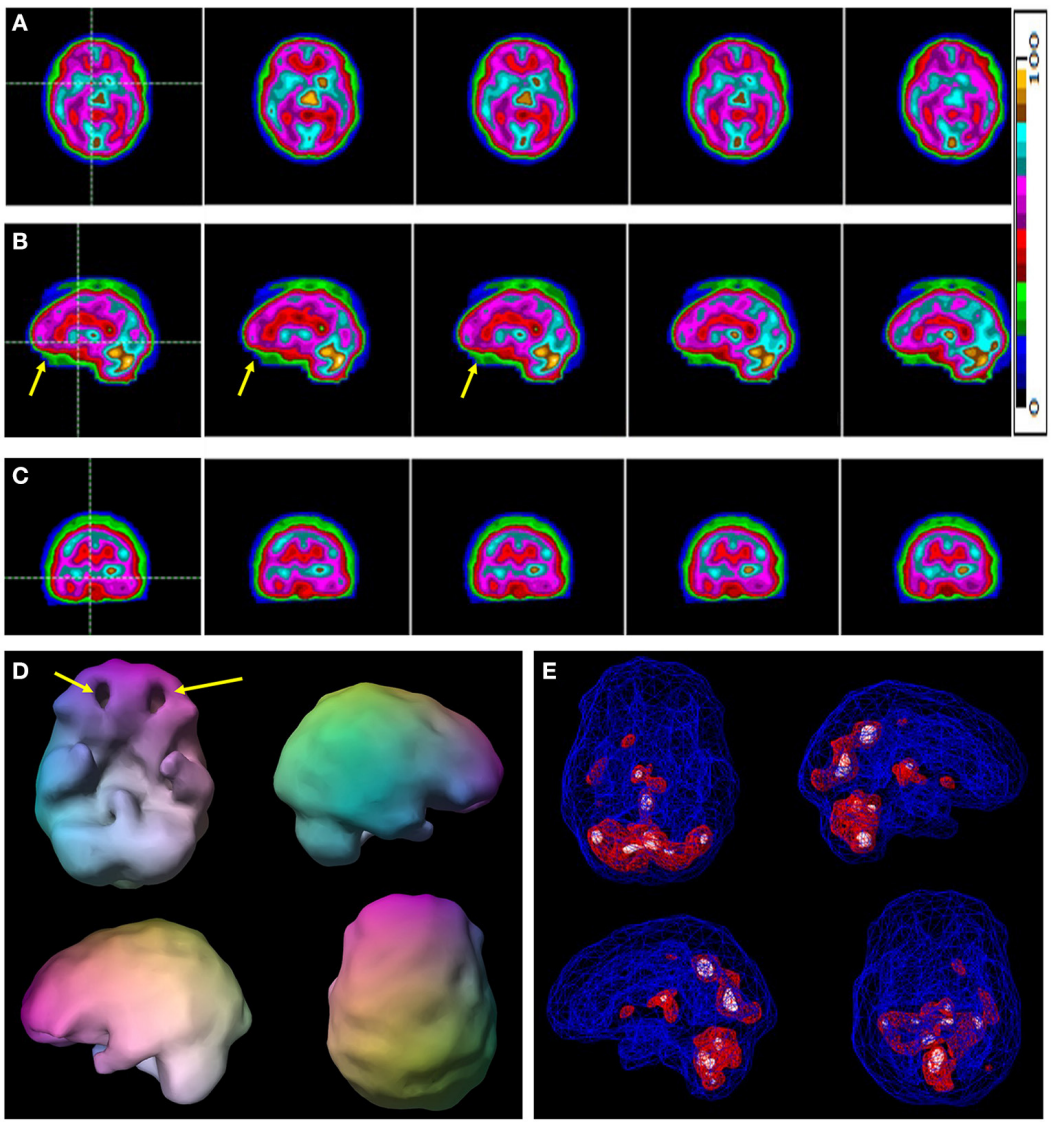
A typical example of a case of ADHD without comorbidity. Selected tomograms in horizontal **(A)**, sagittal **(B)**, and coronal **(C)** orientation are provided. The color scale is the same as Figure 1 for the tomograms is provided. All voxels are scaled to the brain maximum and assigned each a color gradient based on its percentile of activity. Each color step represents a (not necessarily linear) five-percentile-point change in rCBF. Yellow arrows indicate areas of hypoperfusion in the orbitofrontal cortices. **(D)** A 3-D representation of the scan data is shown. The surface setting is the same as in Figure 1. Yellow arrows point to the areas of decreased perfusion in the orbitofrontal cortices. **(E)** A wireframe brain representation is shown with setting the same as in Figure 1.

The results of the logistic regression models with age, gender, and race as co-variates, computation of odds ratios and predicted probabilities with correction for multiple comparisons were input for a ROC analysis which yielded area under the curve (AUC) calculations. The AUC for Visually Read ROI's of the Baseline scan was 97.6%. The AUC for the *post-hoc* ROI analysis of the Baseline scan was 100%. Sensitivity of the visual reads was 100% while specificity was >97%. The sensitivity and specificity of the *post-hoc* ROI analysis were both 100%. Figure 1 is a typical baseline tomogram presentation of the SPECT scan data from a control case. Figure 2 is a typical baseline tomogram of the SPECT scan data from a patient in Group 1 illustrating hypoperfusion by visual interpretation in the medial anterior prefrontal (orbitofrontal) cortex, bilateral temporal lobes, and the anterior cingulate gyri. These findings proved strongly predictive of the diagnosis of ADHD, as illustrated in Table 2. Areas identified by visual read as having highly significant Odds Ratio (*p* < 0.001) for discriminating ADHD from control included: medial anterior prefrontal cortex, left anterior temporal lobe, and right insular cortex. Areas with moderately significant Odds Ratio (*p* < 0.03) included: right lateral middle temporal lobe, right medial temporal lobe, dorsal anterior cingulate gyrus, the genu of the anterior cingulate gyrus, and the left parietal cortex (see Table 2).

**Table 2:** Predictive visually interpreted ROIs from Group 1.

**Brain region**	**Statistical output**
**Baseline**	**Odds ratio of increased probability for ADHD, *p*-value**
Medial anterior prefrontal cortex	5.4, 0.001
Left anterior temporal lobe	4.7, 0.001
Right insular cortex	4.3, 0.001
Right lateral middle temporal lobe	3, 0.01
Right medial temporal lobe	3.6, 0.02
Dorsal anterior cingulate gyrus	2.8, 0.02
Genu anterior cingulate gyrus	4.5, 0.03
Left parietal lobe	4.4, 0.03

Table 3 shows the *post-hoc* quantified ROI regions that were most predictive in distinguishing cases of ADHD in Group 1 from controls. In particular, cerebellar subregions were very predictive (*p* < 0.001), along with the bilateral anterior inferior temporal lobe, bilateral middle temporal pole, and the bilateral inferior occipital lobe. Areas with significant predictive findings (*p* < 0.03) included: bilateral anterior cingulate gyri, bilateral mid orbital frontal cortices, right superior orbital frontal cortex, bilateral hippocampi, right middle occipital cortex, bilateral thalamus, and bilateral pallidum. Some left/right differences were observed. The left anterior cingulate showed a higher odds ratio than the right, while the right orbital frontal cortical areas had higher odds ratios than the corresponding areas on the left.

**Table 3:** Group 1 ROI differences between ADHD and normal.

**Region**	**Control**	**ADHD**	***F*, *p*-value**
**Baseline**			
L caudate	55.6 ± 8.2	53.4 ± 8.1	3.9, 0.04
R caudate	55.7 ± 8.1	53.5 ± 8	4.2, 0.04
L cerebellum 7b	47.6 ± 6.9	54.8 ± 9.4	33.2, <0.001
R cerebellum 7b	47.7 ± 8.1	54.2 ± 10.1	23.5, <0.001
L cerebellum 8	49.2 ± 7.8	55.7 ± 9.1	27.3, <0.001
R cerebellum 8	49 ± 8	55.2 ± 8.9	26.3, <0.001
L cerebellum 9	51.4 ± 8.5	56 ±8.6	15.5, <0.001
R cerebellum 9	51.4 ± 8.2	55.9 ± 8.6	14.9, <0.001
L cerebellum crus1	51.3 ± 8	54.6 ± 8.1	8.7, 0.003
R cerebellum crus1	50.7 ± 8.3	54.6 ± 8.4	11.2, 0.001
L cerebellum crus2	47.1 ± 7.8	54.3 ± 9.5	32.2, <0.001
R cerebellum crus2	47.2 ± 7.8	54.1 ± 9.8	27.3, <0.001
L anterior cingulate gyrus	56.1 ± 8.7	53.1 ± 8.2	7.1, 0.008
R anterior cingulate gyrus	55.7 ± 8.7	53.1 ± 8.2	5.4, 0.02
L mid orbital frontal 9	51.3 ± 9.2	54.2 ± 8.2	6.1, 0.01
R mid orbital frontal 10	50.3 ± 9.3	54.1 ± 8.5	10.1, 0.002
R superior orbital frontal lobe 10	51.8 ± 9.2	54.3 ± 7.9	5.1, 0.02
L hippocampus	56.2 ± 8.6	53.7 ± 8.1	4.9, 0.02
R hippocampus	56.2 ± 8.5	53.7 ± 7.8	5.5, 0.01
R insula	55.6 ± 8.2	53.3 ± 7.9	4.3, 0.04
L inferior occipital lobe	49.9 ± 8.5	54.7 ± 8.6	16.2, <0.001
R inferior occipital lobe	48.6 ± 9.3	54.9 ± 8.8	26.8, <0.001
R middle occipital lobe	51.1 ± 8.7	54.7 ± 8.3	10.3, 0.001
L superior occipital lobe	51.9 ± 8.5	54.4 ± 8.2	4.6, 0.03
R superior occipital lobe	52.1 ± 8.2	54.5 ± 8.1	4.3, 0.04
L pallidum	56.2 ± 8.6	53.5 ± 8.2	5.6, 0.01
R pallidum	56.3 ± 8.7	53.8 ± 8.1	4.6, 0.03
R superior parietal	49.6 ± 8.4	52.4 ± 8.7	5.3, 0.02
L anterior inferior temporal lobe	47.9 ± 9.2	53.4 ± 9.3	18.3, <0.001
R anterior inferior temporal lobe	48 ± 7.6	53.4 ± 9.7	17.1, <0.001
L mid inferior temporal lobe	51 ± 8.3	54.1 ± 8.4	7.5, 0.006
R mid inferior temporal lobe	50.5 ± 7.6	53.9 ± 8.2	9.3, 0.002
L posterior inferior temporal lobe	51.5 ± 7.8	54.3 ± 8.1	6.4, 0.01
R posterior inferior temporal lobe	51 ± 8.5	54.6 ± 8.2	9.5, 0.002
L mid temporal pole	49.7 ± 9.1	53.7 ± 9.2	10.2, 0.001
R mid temporal pole	48.6 ± 9.2	53.9 ± 9.3	17.1, <0.001
L thalamus	55.1 ± 6.4	53.1 ± 7.9	5.1, 0.02
R thalamus	54.9 ± 7.1	53.4 ± 7.8	5.2, 0.02
Vermis 10	56.5 ± 6.9	53 ± 8.1	10.3, 0.001
Vermis 8	51 ± 7.1	55.2 ± 8.2	11.2, 0.001

*This table shows the quantified statistically significant ROI differences between ADHD and normal controls in Group 1 on baseline and concentration scans*.

## Discussion

In the adult population, there was high separation between non-comorbid ADHD patients vs. healthy controls using this method. The ROC characteristics were similar for the baseline scan data whether using visual or quantitative analysis. Since the *post-hoc* ROIs were not used in any way to initially establish the clinical diagnoses, they serve as a particularly rigorous independent predictor of diagnostic category. However, it is emphasized that nuclear medicine physicians and radiologists typically use visual analysis for readings SPECT scans in a clinical setting; thus the high sensitivity and specificity of the identified areas serve as promising steps for the translation of SPECT markers for ADHD more widely into clinical practice.

To guide the relevance of these results as possible ADHD biomarkers we use the term *biomarker* as defined by the FDA BEST criteria (16) - “a defined characteristic that is measured as an indicator of normal biological processes, pathogenic processes, or responses to an exposure or intervention, including therapeutic interventions.” A safe and effective biomarker for ADHD could guide diagnosis and treatment. Treating ADHD based solely on clinical indications is not without risk. For example, the differentiation of ADHD from incipient bipolar disorder is challenging. Clinical experience and research data have shown that stimulant medications can precipitate a manic episode, exacerbate mood instability, and/or increase rapid cycling. Some (54) hypothesize that stimulant medication exposure can permanently alter the course of bipolar disorder in some children. In addition, atomoxetine, FDA-approved for the treatment of ADHD, has been clinically found to be a potent mood destabilizer. A large open-label naturalistic case series (55) found that roughly 33% of patients became mood dysregulated on atomoxetine. Symptoms of aggression, hypomania, agitation, and frank mania were reported in patients, some of whom lacked any previous history of mood symptoms. Thus, correctly differentiating ADHD from incipient bipolar disorder and/or possible variants of ADHD who show adverse reactions to stimulants and/or atomoxetine would mitigate serious patient harm.

Prior SPECT neuroimaging studies of ADHD in children and adults have varied in quality considerably, but have consistently pointed in the direction of potential biomarkers. The first baseline investigations by Lou et al. (56, 57) utilized Xenon-133, which provides a single-pass perfusion scan and absolute quantification of perfusion in units of ml/min/100 g of tissue, albeit with limited resolution. In addition, confounds of methodology and diagnostics further limit the validity of these studies. Nevertheless, a later study by the same group with better technology replicated the findings of decreased perfusion of the striatum based on Xenon-133 quantitative perfusion (58). Gustafsson et al. (59) compared baseline SPECT scan data to EEG and neurological examination in a group of 28 children with broadly defined ADHD (based on Conners Parent Rating Scale and Wechsler Intelligence Scale for Children). While this study lacked a control group, it is notable for its correlation of EEG findings, symptoms, soft neurological signs, and functional neuroimaging. The key findings were that patients with ADHD showed decreased frontal lobe perfusion and the degree of symptoms correlated with the degree of hypoperfusion (decreased activity) in the frontal lobes (59). In addition, patients with ADHD had a number of soft neurological signs and physical anomalies. Similarly, Spalletta et al. (60) found a correlation between symptoms of ADHD and frontal lobe perfusion. In a carefully screened group of 8 children (inclusion criteria were medication-naïve, normal MRI scan, no comorbid psychiatric diagnoses, IQ > 80, and ADHD diagnosis based on Stroop Test and neurometric data), baseline scans done under sedation showed decreased perfusion of the left dorsolateral prefrontal cortex and orbital frontal cortex. Relatively increased perfusion in the right prefrontal cortex and relatively decreased perfusion in the left prefrontal cortex were correlated with worse clinical symptomatology (60). Given that the radiopharmaceuticals HMPAO and ECD are distributed and relatively fixed within 2 min of injection and a waiting period of roughly 15–30 min was allowed after tracer injection, sedation during the scan would have no significant effect on the distribution of radiotracer and scan results.

Cognitive challenge tasks highlight the deficits in task-specific function that characterize ADHD. A response inhibition task was administered to 20 children with ADHD and 4 controls (61). The authors concluded that children with ADHD exhibited a right prefrontal cortex dysfunction based on an exaggerated left-to-right asymmetry of perfusion.

Medication response trials using SPECT or fMRI can also point toward areas which may serve as candidate imaging biomarkers. In general, control patients showed equivocal differences in perfusion in the frontal lobes whether they were on- and off-stimulant medications. In contrast, children with ADHD demonstrate decreased perfusion at baseline (which equates to decreased activity) in the frontal cortices and often the temporal lobes and cerebellum (42, 61–66) that increased with stimulant medication (61, 63, 64, 67).

Kim et al. (68) conducted an elegant treatment effect study involving 40 medication-naïve children with ADHD (who were evaluated with ADHD assessment scales, structured clinical interviews, and neuropsychological testing, and had normal MRI or CT scans) and who were compared to 17 controls using statistical parametric analysis before and after treatment with methylphenidate (68). Baseline HMPAO SPECT scans were obtained in 40 children (age 9.7 ± 2.1 years) diagnosed with ADHD. Strict exclusion criteria eliminated subjects with IQ below 90, learning disorders, neurological disorders, or comorbid psychiatric diagnoses of mood, anxiety, or conduct. These baseline scans were then compared statistically to baseline scans of 17 similar strictly screened controls (age 10.5 ± 2.2 years). Then, ADHD subjects were started on methylphenidate at standard doses (0.3–1.0 mg/kg/day). After 4–5 weeks of stimulant treatment, ADHD subjects underwent a second baseline SPECT scan while taking methylphenidate. The second scan was again compared statistically to the control scans and differences between the pre- and post-medication scans were identified. All ADHD subjects showed significant improvement in symptoms based on psychometric testing while taking methylphenidate. Pre-treatment scans of subjects with ADHD showed decreased perfusion of the prefrontal cortex and middle temporal gyri but showed increased perfusion in the somatosensory cortex and anterior cingulate gyri, compared to controls. After treatment with methylphenidate, ADHD subjects showed increased perfusion of the prefrontal cortex relative to their own pre-medication scans. Perfusion in the somatosensory cortex and striatum was reduced (68). 3D SPECT images in ADHD have also been used by Schneider et al. (43, 69) to show orbitofrontal cortex hypoperfusion in patients with ADHD.

Lorberboym et al. (70) examined ADHD with and without comorbid learning or behavioral diagnoses (oppositional defiant disorder, conduct disorder, learning disorder, mood disorder). After psychometric testing and structured clinical interview, a group of 8 children with simple ADHD, a group of 11 children with ADHD comorbid for one or more of the above diagnoses, and a group of 9 age-matched controls underwent SPECT scanning, and the scans were compared. Using a semi-quantitative analysis of selected regions of baseline scans, ~50% of cases showed decreased frontal lobe perfusion, but all of the cases who were comorbid demonstrated decreased temporal lobe perfusion (70). These findings were largely confirmed in a study of 19 children with specific learning disorders compared to 12 children with ADHD (71). Unfortunately, no control group was included in this confirmatory study. Children with learning disorders showed relatively decreased perfusion in the temporal lobes and the right parietal lobe, but also in the bilateral basal ganglia.

Recently, these neuroimaging findings have been replicated using a newer technique of infrared spectroscopy (72). A sample of 150 children with ADHD were compared to 51 controls in a series of concentration tasks. Children with ADHD demonstrated low activation of the medial prefrontal (orbitofrontal) cortex during vigilance and concentration tasks.

## Comorbidity Is the Rule, Not the Exception

Unfortunately, comorbidity is the rule, rather than the exception, in cases of ADHD. Therefore, a study of pure ADHD is not sufficient to identify a clinically useful imaging biomarker for ADHD. Children with ADHD frequently have comorbid anxiety, oppositional disorders, or learning disorders (73, 74). For example, learning disorders are highly prevalent among those with ADHD, ranging from 10 to 90% comorbidity (75, 76). The prevalence of anxiety disorders among those with ADHD ranges from 15 to 35% (77, 78). Depressive disorders occur in 12–59% of children with ADHD (79, 80). The prevalence of conduct disorder and oppositional defiant disorder among those with ADHD ranges from 30 to 50% (76, 81). The comprehensive Multimodal Treatment Study of Children with Attention-Deficit/Hyperactivity Disorder (MTA) study similarly found 29 % of children diagnosed with ADHD were comorbid for either conduct disorder or oppositional defiant disorder (78). These comorbidities profoundly alter the clinical picture of ADHD and undoubtedly alter the response to pharmacological interventions. Current diagnostic methods fail to fully assess the presence and impact of comorbidities.

ADHD in adults was largely unrecognized prior to 2002 (82, 83). Comorbidity clouds the diagnosis in adults, as well. An estimated 65–89% of adult patients with ADHD also have anxiety disorders, depressive disorders, bipolar disorder, personality disorders, drug abuse, and alcohol abuse (84). Comorbidity of ADHD and depressive disorders ranges from 18.6 to 53% (85). Anxiety disorders are quite common in adults with ADHD, approaching 50% comorbidity (86). An estimated 20–47% of adults with ADHD are comorbid for bipolar disorder (87, 88). Comorbid substance abuse is estimated at 23.3% based on a systematic review (89). Although not well-studied, at least one analysis of insurance data indicates that ADHD leads to an increased risk of TBI. The risk for receiving any form of TBI was 9.8% for those with ADHD compared to 2.2% for those without ADHD, representing a 4.5-fold increase in risk (90).

## Next Steps

Herein, we have analyzed baseline SPECT scans of adult patients with pure ADHD compared to an age-matched control group. Interesting potential candidate neuroimaging biomarkers have been identified. Next, it will be vital to expand these analyses to patients with ADHD comorbid for diagnoses such as anxiety, depression, bipolar disorder, addictions, gender-based differences, and/or brain trauma (among others). Going forward, we will also implement machine learning algorithms to rigorously test the candidate biomarkers utilizing our large-N dataset. These algorithms will be applied to our adolescent datasets to explicitly address the complex diagnostic picture of ADHD symptomatology in the adolescent patient.

## Conclusions

Strengths of this study include the large sample size of non-comorbid ADHD compared to a well-characterized control group, the consistent methods of visual interpretation of rest (or baseline) scans with a well-validated functional imaging modality, and detailed quantitative analysis. The study is further enhanced by a *post-hoc* ROI analysis which had similar findings. That said, several caveats of the study must be addressed. First, the data are retrospective, and higher levels of evidence can be derived from either prospective studies or randomized clinical trials. However, the large sample size and diverse multi-site study optimizes the generalizability of our results. Second, this dataset does not have associated structural imaging data. Such information would have been useful in characterizing any atrophy associated hypoperfusion. However, the use of functional neuroimaging is essential in characterizing subtle abnormalities that may not be apparent on even quantitative structural neuroimaging. Third, in such a large retrospective database, there are many patients who were on one or more medications for medical and psychiatric indications. While known stimulants and depressants of brain function were withheld prior to scanning, the confounding effects of medical and psychiatric medications cannot be completely eliminated.

Using the method described above we consistently distinguished adult ADHD patients from healthy controls. Given the wide availability of brain SPECT imaging and the need for accurate diagnosis in ADHD, this test, with appropriate reader training, could provide valuable information in clinical practice.

## Data Availability Statement

The original contributions presented in the study are included in the article/Supplementary Material. Further inquiries can be directed to the corresponding author/s.

## Ethics Statement

The studies involving human participants were reviewed and approved by IntegReview (http://www.integreview.com/) and Western IRB (WIRB # 20021714). The patients/participants provided their written informed consent to participate in this study.

## Author Contributions

All authors listed have made a substantial, direct, and intellectual contribution to the work and approved it for publication.

## Conflict of Interest

TH is the president and principal owner of the Synaptic Space, a neuroimaging consulting firm. He is also CEO and Chairman of the Board of Neuro-Luminance Corporation, a medical service company. He is also president and principal owner of Dr. Theodore Henderson, Inc., a medical service company. He is also Vice-President of the Neuro-Laser Foundation, a non-profit organization. He is a member of and a former officer of the Brain Imaging Council Board of the Society of Nuclear Medicine and Molecular Imaging (SNMMI). Since 2017, he has served in the SNMMI Brain Imaging Outreach Working Group. Currently, he serves as president of the International Society of Applied Neuroimaging. TH has no ownership in, and receives no remuneration from, any neuroimaging company. No more than 5% of his income is derived from neuroimaging. DA is the sole owner of Amen Clinics, a group of nine neuropsychiatric clinics that perform brain SPECT imaging. The remaining author declares that the research was conducted in the absence of any commercial or financial relationships that could be construed as a potential conflict of interest.

## Publisher's Note

All claims expressed in this article are solely those of the authors and do not necessarily represent those of their affiliated organizations, or those of the publisher, the editors and the reviewers. Any product that may be evaluated in this article, or claim that may be made by its manufacturer, is not guaranteed or endorsed by the publisher.
